# *Aggregatibacter actinomycetemcomitans* osteomyelitis in a 12 year old boy: case report emphasizing the importance of tissue culture, and review of literature

**DOI:** 10.1186/s12941-017-0186-0

**Published:** 2017-03-14

**Authors:** Ketaki Sharma, Poonam Mudgil, John S. Whitehall, Iain Gosbell

**Affiliations:** 1 Department of Paediatrics, School of Medicine, Western Sydney University, Sydney, NSW Australia; 2 Department of Infectious Diseases and Microbiology, School of Medicine, Western Sydney University, Sydney, NSW Australia

## Abstract

**Background:**

*Aggregatibacter actinomycetemcomitans* most commonly causes periodontitis but has been reported to infect heart valves, soft tissue, brain and lungs, and distal bones. Osteomyelitis distal to the jaw is rarely described.

**Case presentation:**

We report an unusual and rare case of chronic osteomyelitis caused by *A. actinomycetemcomitans* in the toe of a paediatric patient, and review the available literature. The infection was managed with intravenous antibiotics followed by oral antibiotics.

**Conclusion:**

This is an unusual presentation of *A. actinomycetemcomitans* causing chronic osteomyelitis presumed due to nidation in a minimally damaged bone, associated with bacteraemia of an oral commensal. It occurred in the toe, without obvious dental predisposition; associated with minimal clinical disturbance and with muted immune response.

## Background

*Aggregatibacter actinomycetemcomitans*, previously known as *Actinobacillus actinomycetemcomitans*, is a microaerophilic, capnophilic gram negative cocco-bacillus that is an oral commensal in over 30% of apparently healthy children [1]. It is most commonly associated with aggressive periodontitis but that infection may be chronic [2]. Extra-oral infections, presumably due to haematogenous spread from infected periodontium [3], have occurred on heart valves and in soft tissue including brain and lung, joints and distal bones, mostly in adults. We report distal, chronic osteomyelitis in a 12 year old boy.

## Case presentation

An otherwise well, immunocompetent 12 year old boy presented to Campbelltown Hospital, NSW, with a 2 week history of pain, swelling, and purulent discharge from his right great toe. His past dental history is unknown and not recorded in records. He had ‘stubbed’ the toe 12 months previously and mild, intermittent pain had persisted, un-associated with any systemic symptoms or signs of disease. Two weeks before presentation, a purulent discharge had emerged from the side of the toe. He had received a course of oral flucloxacillin from his general practitioner. On examination, there was superficial crusting on the medial aspect of the right great toe associated with purulent discharge. There was mild swelling around the inter-phalangeal joint but no erythema and tenderness, and there was full and painless range of movement throughout the foot. There were no abnormalities found on general examination of the child, and his teeth and gums appeared normal on inspection of his mouth. Subsequent dental review revealed no evidence of periodontitis.

**Fig. 1:**
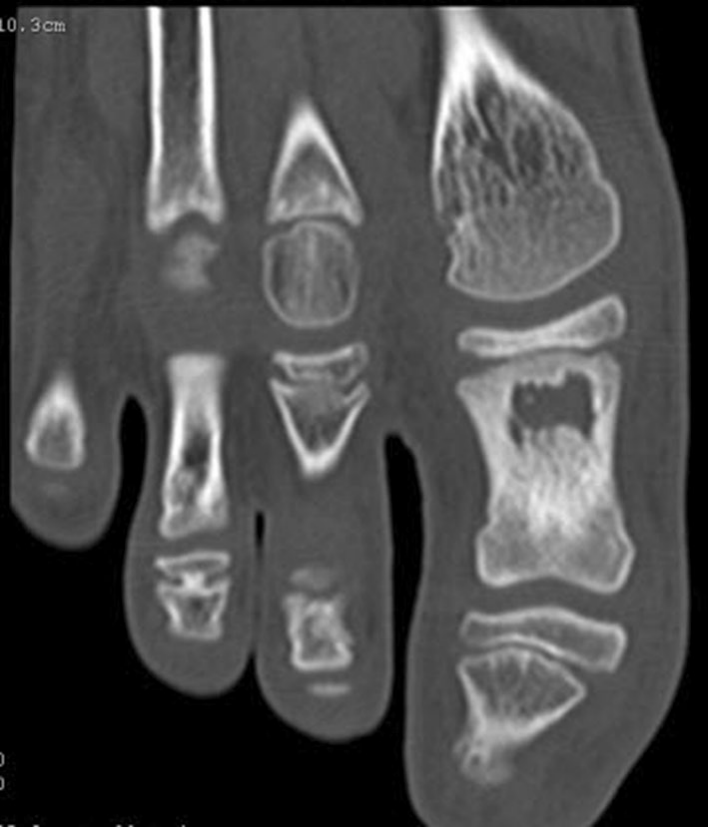
X-ray image showing lytic changes of osteomyelitis in the proximal phalanx of the big toe

Investigations revealed a normal white cell count (total 6.2 × 10^9^/L; neutrophils 1.96 × 10^9^/L), un-elevated levels of inflammatory markers (erythrocyte sedimentation rate 2 mm/h, C-reactive protein < 0.4 mg/L), and both gram positive cocci and gram negative bacilli in a wound swab whose culture failed to grow organisms. An X-ray revealed a lytic lesion in the proximal phalanx of the great toe while bone scan confirmed cystic changes, sclerosis and hyperaemia (Fig. 1).

At operation, a sinus was found passing through organised purulent material to the infected proximal end of the phalanx. There was no sequestered bone. After 6 days, culture of curetted bone grew *A. actinomycetemcomitans* which was sensitive to ampicillin and cefotaxime. According to the Sydney South West Pathology Service tissue protocol, culturing is done on horse blood, chocolate, MacConkey, Columbia colistin nalidixic acid (CAN), and anaerobic agars, and thioglycollate broth. Incubation is done at 37 °C and read daily for up to 7 days before reported as final culture negative. A blood culture had been taken at presentation and was negative at 5 days.

His treatment had begun with intravenous flucloxacillin and clindamycin but the latter was ceased and cefotaxime started when culture results became available. A total of 7 days of intravenous therapy was followed by 12 weeks of oral amoxicillin during which the infection resolved. Given the known association of *A. actinomycetemcomitans* infections with endocarditis, an echocardiogram was performed but no abnormalities were detected.

## Discussion

*Aggregatibacter actinomycetemcomitans* is a member of the *Haemophilus aphrophilus* now named *Aggregatibacter aphrophilus*, *Actinobacillus actinomycetemcomitans, Cardiobacterium hominis, Eikenella corrodens* and *Kingella* spp. (HACEK) group of gram negative bacteria characterized by fastidious, capnophilic growth requirements. *Kingella kingae* is a well-recognised albeit rare cause of skeletal infections in children. Conversely, *A. actinomycetemcomitans* is noted for its association with both aggressive and chronic periodontal infection, as well as for rare infection of heart valves and soft tissue including brain and lungs. While infection of the bone of the jaw may be an extension of periodontitis, osteomyelitis of distal bones is most rare, and usually involves the vertebrae.

A medline bibliographic search was conducted to identify documented cases of distal osteomyelitis caused by *A. actinomycetemcomitans*, in both its recent and former names. Data was collected to include age, sex, clinical presentation, time to diagnosis, site of infection, laboratory findings and predisposing factors and these details together with those of our patient are presented in Table 1.

**Table 1:** Clinical features of reported cases of *Aggregatibacter actinomycetemcomitans* osteomyelitis and septic arthritis

Case no. (Ref)	Age/sex of patient	Infectious syndrome (additional pathogens)	Symptoms	Duration of symptoms	Predisposing factors	Inflammatory markers	Diagnosis	Management
1 [15]	38 years male	Osteomyelitis of mandible	Pus discharge from mandible afebrile	20 days	Mandibular fracture 2 months prior	Not reported	Culture of pus	Debridement Ampicillin Tetracycline
2 [16]	45 years male	Vertebral osteomyelitis	Back pain, axillary lymphadenitis afebrile	2 months	Dental caries	WCC normal ESR 31, 45 mm/h	Culture of bone biopsy	Ampicillin 6/52
3 [17]	72 years male	Vertebral osteomyelitis and epidural abscess	Lower limb weakness, urinary retention, fever	2 days	Severe periodontal disease	WCC 12.6 × 10^9^/L ESR 90 mm/h CRP 27.7 mg/L	Culture of pus	Ceftriaxone 6/52
4 [18]	16 years female	Vertebral osteomyelitis, mediastinal and pulmonary abscesses (*Actinomyces isralii, B. corodens)*	Not reported	7 months	Dental caries, teeth extractions	Not reported	Not reported	Penicillin 4/52
5 [19]	47 years male	Osteomyelitis of tibia, lung abscess, skin abscesses (*Actinomyces meyeri)*	Knee effusion, subcutaneous nodules, fever	3 weeks	Caries, dental stumps	WCC 13.3 × 10^9^/L ESR 117	Biopsy of subcutaneous abscess	Debridement, penicillin 1 year
6 [12]	66 years male	Vertebral osteomyelitis (organism intermediate between *A. actinomycetemcomitans* and *Haemophilus aphrophilus)*	Lumbar pain, sciatica, rigors	4 days	Nil	WCC 12.7 × 10^9^/L ESR 84 mm/h	Culture of bone biopsy	Flucloxacillin 4/52, cefotaxime 4/52, amoxicillin 4/52
7 [13]	Adult male	Chronic osteitis of fifth metacarpal, associated drainage sinus *(pure growth A. actinomycetemcomitans)*	Recurrent abscesses after tooth was surgically removed from hand	6 months	Tooth embedded in hand during an assault	Not reported	Culture of pus	Debridement Ampicillin
8 [20]	45 years male	Chronic osteomyelitis of distal femur, septic arthritis	Pain and swelling of medial aspect of knee	3 months	Open fracture of knee 20 years prior	WCC 8.2 × 10^9^/L	Culture of synovial fluid. Bone culture negative	Debridement Cefotaxime Amoxicillin
9 [our patient]	12 years male	Osteomyelitis of proximal phalanx of the great toe	Intermittent toe pain 2/52 discharging pus	12 months	Nil	WCC 6.4 × 10^9^/L CRP < 0.4 mg/L	Culture of bone biopsy	Debridement Cefotaxime 1/52 Amoxicillin 12/52

There were seven bone infections due to *A. actinomycetemcomitans*, and one with an organism apparently intermediate between it and *Haemophilus aphrophilus*. In two of these eight cases, there was co-infection of *A. actinomycetemcomitans* with other bacteria, which is not unusual [4]. The age of the patients ranged from 16 to 78 years; seven were male.

Four infections involved vertebrae, which is unsurprising given they are the most common site of acute haematogenous osteomyelitis in adults. Six infections were associated with dental disease or oral trauma: four had dental disease, one a mandibular fracture, and one had an opponent’s tooth embedded in his hand. One patient had septic arthritis of the knee and chronic osteomyelitis of the distal femur, but had had an open fracture of the knee 20 years previously.

Duration of symptoms ranged from 2 days to 7 months, but three of the infections were classified as chronic osteomyelitis. The white cell count was normal to barely raised in all cases, but inflammatory markers were elevated in all, except our patient. Resistance to antibiotics was not reported.

The infectivity of *A. actinomycetemcomitans* is due to many virulence factors that modulate host immunity, facilitate invasion and retard repair. Exotoxins include a leukotoxin (LtxA) which promotes lysis of defending macrophages, mast cells and leukocytes, and a cytolethal distending toxin (CDT) which promotes death of host cells by restricting their proliferation while encouraging osteoclastogenesis. The exotoxin leukotoxin A combines with endotoxins to induce inflammation with subsequent bone resorption [3, 5]. Defence of the organism is aided by the formation of biofilms with synergistic organisms in the protected crevice between the gums and the teeth. The film can attach to the teeth and be nourished by a continuous efflux of fluid derived from blood plasma and containing amino acids, peptides and proteins [6].

*Aggregatibacter actinomycetemcomitans* has been found in the mouths of >1/3 of apparently healthy children and is believed to travel horizontally and vertically within families, presumably spread in saliva [7, 8]. A recently reported case of chronic wound infection suggests an exogenous origin [9] but, otherwise, infections distal to the mouth appear to be endogenous and blood borne. Periodontal disease is believed to predispose to such spread. It should be noted that periodontal disease (gingivitis associated with loss of the tooth-supporting tissues, alveolar bone and connective tissues) may not be associated with marked evidence of dental caries. Indeed, a negative relationship has been described [10].

Osteomyelitis in hands and feet is unusual in children unless associated with dactylitis of a haemoglobinopathy in which cases there is acute onset with pain, swelling and, usually, systemic symptoms associated with salmonellae infection [11]. Chronic, symptom-minimal infection is rare but may occur with tuberculosis [12].

We can only postulate about the lack of aggression by *A. actinomycetemcomitans* and the muted immune response in our patient: perhaps the organism entered a sessile state of biofilm existence while deprived of nutritionally rich gingival crevicular fluid [13] and also deprived of synergistic relationships with other oral commensals [14].

Knowing that most bone infections in children are due to *S. aureus*, treatment of our patient was started with flucloxacillin, which was continued for 1 week. After isolation of *A. actinomycetemcomitans*, the flucloxacillin was changed to amoxicillin, since HACEK organisms are not susceptible to flucloxacillin, and the isolate was found to be susceptible to amoxicillin The gram positive cocci and gram negative rods seen on gram stain from the wound swab did not grow in the blood culture, and may have been anaerobes, which are commonly found with organisms such as *A. actinomycetemcomitans*. Alternatively the prior antibiotics from the family doctor might have inhibited the growth of bacteria. Resistance to amoxicillin has been reported in 77% of distal infections or cultures of *A. actinomycetemcomitans* derived from the mouth, though sensitivity to amoxicillin/clavulanic acid is expected [15].

## Conclusion

We have described an unusual presentation of *A. actinomycetemcomitans* osteomyelitis presumed due to nidation in a minimally damaged bone, associated with bacteraemia of an oral commensal. It occurred in the big toe of a young, otherwise healthy child without obvious dental predisposition; was chronic and associated with minimal clinical disturbance until a sinus opened to the skin; was unassociated with alteration in white cell count and inflammatory markers and was sensitive to amoxicillin. The peripheral wound swab did not grow *A. actinomycetemcomitans.* Representative specimens are important, especially if blood cultures are negative and/or there is failure of empirical treatment. Surface swabs can be misleading, and deep specimens obtained by needle or surgery are preferred to diagnose the cause of bone infection.
